# Fetal and Neonatal Heart Rate Trends in Preterm Delivery: A Clinical Study from the Week before to the Week after Birth

**DOI:** 10.1055/a-2806-2923

**Published:** 2026-02-23

**Authors:** Chantal Eenkhoorn, Tom G. Goos, Arie Franx, Jenny Dankelman, H. Rob Taal, Sten P. Willemsen, Alex J. Eggink

**Affiliations:** 1 Department of Obstetrics and Gynecology, Erasmus MC, University Medical Center, Rotterdam, The Netherlands; 2 Department of Neonatal and Pediatrics Intensive Care, Erasmus MC, University Medical Center, Rotterdam, The Netherlands; 3 Department of Biomechanical Engineering, Delft University of Technology, Delft, The Netherlands; 4 Department of Biostatistics, Erasmus MC, University Medical Center, Rotterdam, The Netherlands

**Keywords:** fetal heart rate, neonatal heart rate, fetal–neonatal transition, generalized estimating equation model

## Abstract

**Objective:**

This study aimed to explore the fetal heart rate trend in the week before birth, the transition from fetal to neonatal heart rate, and the neonatal heart rate trend in the week after birth in preterm neonates admitted to a tertiary care hospital, considering maternal and neonatal factors.

**Study Design:**

A retrospective cohort study was conducted, including neonates born between 24 and 34 weeks of gestation. Baseline heart rate, average deceleration capacity, standard deviation, skewness, and sample entropy were assessed using interrupted time series and difference-in-differences analyses. Subgroup analyses were performed according to gestational age at birth, sex, birth weight, mode of delivery, Apgar score at 5 minutes, umbilical cord pH, and neonatal medication.

**Results:**

The fetal and neonatal heart rate of 123 patients was analyzed. After birth, step change of baseline (2.23 bpm, *p* < 0.05), average deceleration capacity (0.43 bpm, *p* < 0.001), and skewness (0.47 nu, *p* < 0.001) increased, while sample entropy (−0.68 bits, *p* < 0.001) and standard deviation (−1.15 bpm, *p* < 0.001) decreased. Postnatally, baseline increased in linear slope and decreased in quadratic slope (both *p* < 0.001). Average deceleration capacity decreased in linear slope ( *p* < 0.001). Sample entropy and standard deviation increased in linear slopes (both *p* < 0.001). Skewness increased in quadratic slope ( *p* < 0.05). Subgroup analyses revealed that delivery mode, medication, and birth weight modulated these trends.

**Conclusion:**

This study provides unique insights into heart rate frequency and variability trends during the period around preterm birth. It highlights the dynamic physiological adaptation that occurs during the transition from intrauterine to extrauterine life in preterm infants and may help inform future research on fetal and neonatal monitoring and clinical management.

**Key Points:**

- Heart rate frequency and variability dynamics were assessed around preterm birth in a NICU cohort.
- After birth, heart rate frequency increased while variability measures decreased.
- Heart rate frequency and variability differed by gestational age, birth weight, medication, and delivery mode.

## Introduction

Heart rate monitoring is a critical aspect of fetal and neonatal care, as it gives insights into the cardiovascular function and clinical condition of both the fetus and neonate. In the fetal period, heart rate monitoring provides information on the oxygenation and maturation of the autonomic nervous system of the fetus, with heart rate variability serving as an important indicator of fetal distress. 1 2 Abnormal heart rate patterns may indicate underlying problems such as hypoxia or infection. 3 4 In the neonatal period, continuous heart rate monitoring provides information on the adaptation to extrauterine life and cardiovascular function of the neonate. Variations in heart rate could reflect early signs of distress caused by, for example, sepsis, necrotizing enterocolitis, or long-term morbidity. 5 6 7 8 9 Heart rate monitoring in both the fetal and neonatal period is crucial for detecting early signs of deterioration, guiding clinical interventions, and improving health outcomes for both the fetus and the neonate.

In clinical practice, the fetal heart rate is conventionally measured noninvasively using Doppler ultrasound. A Doppler ultrasound probe placed on the abdomen of the mother measures the heart rate by detecting the movement of the fetal heart and blood flow. Fetal heart rate monitoring in clinical conditions is utilized intermittently, for a minimum of 30 minutes per day, or continuously during labor. At the neonatal intensive care unit, the neonatal heart rate is continuously measured using electrocardiography. Electrodes placed on the neonates body measure the electrical activity of the heart.

Fetal to neonatal transition involves significant physiological changes in the cardiovascular system. Adaptation to the extrauterine life includes adjustments in the circulatory system to support lung function of the neonate. These include the cessation of uteroplacental circulation, a decrease in pulmonary vascular resistance, an increase in systemic vascular resistance, and the functional closure of the fetal shunts. 10 Monitoring heart rate trends during the intrauterine to extrauterine transition provides insight on the dynamical adaptations of the heart rate and may be essential for identifying possible abnormalities. 11 12

The objective of this study is to explore (1) the trend of the fetal heart rate in the week before birth, (2) the transition from fetal-to-neonatal heart rate, and (3) the trend of the neonatal heart rate in the first week after birth, in a cohort of prematurely born babies who were admitted to a tertiary care hospital, considering maternal and neonatal factors.

## Materials and Methods

A retrospective cohort study was conducted to analyze heart rate measurements of fetuses and neonates admitted to a tertiary care hospital (Erasmus MC Sophia Children's Hospital, Rotterdam, the Netherlands). Women admitted between July 1, 2017 and December 31, 2021 were screened for eligibility. Women included in the study were those who delivered at the hospital between 24 and 34 weeks of gestation, underwent fetal heart rate monitoring during the week prior to delivery, had their newborn admitted to the neonatal intensive care unit immediately after birth, and whose newborn underwent heart rate monitoring within the first week after birth. Heart rate data and patient characteristics, including gestational age at birth, sex, birth weight, mode of delivery, Apgar score at 5 minutes, umbilical cord pH, and neonatal medication administration (noradrenaline, dopamine, vasopressin, hydrocortisone, adrenaline, milrinone, dobutamine, alprostadil, enoximone, labetalol, isoprenaline, propofol, morphine, fentanyl, sucrose, midazolam), were obtained from the electronic health records (HiX, Chipsoft, Amsterdam, the Netherlands) and the hospital's neonatal database.

## Signal Source

Fetal heart rate was intermittently monitored with the Avalon FM30 (Royal Philips NV, Amsterdam, The Netherlands). This device employes noninvasive Doppler ultrasound and provides a fetal heart rate value every 0.25 seconds, rounded to the nearest quarter of a beat. In cases of significant signal loss during delivery, a fetal scalp electrode was used to measure heart rate invasively. Neonatal heart rate was continuously monitored with the Infinity-M540 (Dräger Medical, Lübeck, Germany). This device uses electrocardiography and provides a neonatal heart rate value every second, with a measurement range of 15 to 300 beats per minute (BPM) and rounded to the nearest beat. 13 When electrocardiographic electrodes were not used, which is standard practice in neonates less than 26 weeks of gestation at our center, data from the SET pulse oximeter module (Masimo, Irvine, California, United States) connected to the M540 were used. The SET pulse oximeter calculates heart rate from peripheral flow pulse data providing an output every second with a measurement range of 25 to 240 beats per minute and rounded to the nearest beat. 14 Heart rates recorded by the Infinity-M540 and SET pulse oximeter were considered equivalent, based on prior research showing no statistically significant difference between ECG and SET pulse oximeter derived average heart rates in newborns. 15 In both fetuses and neonates, heart rate variability indices were computed from averaged heart rate time series rather than from beat-to-beat RR intervals.

## Preprocessing and Artifact Handling

Data were preprocessed to enhance the heart rate signal. For the fetal heart rate, first the start of the recording was defined as the first value within 20% of the median fetal heart rate during the initial 10 minutes of valid data, ensuring it exceeded 110 bpm to avoid maternal–fetal confusion. Second, fetal heart rate values below 50 bpm or above 220 bpm were considered missing data. Third, potential artifacts were identified by substituting missing data with preceding entries and segmenting the signal when consecutive heart rate values differed by more than 25 bpm. Segments lasting 60 seconds or less, or with a median heart rate differing by more than 25% from the overall median, were flagged as artifacts and replaced with missing values if they differed by more than 20% from the previous segment's median. This process was conducted in both forward and backward directions. Fourth, cubic spline interpolation filled gaps were shorter than 20 seconds, only if the heart rate before and after the gap differed by less than 25 bpm. Lastly, fetal heart rate was down sampled to 1 Hz and rounded to the nearest beat to match the neonatal heart rate data. For neonatal heart rate data, duplicates were removed, heart rate values below 30 bpm and above 240 bpm were treated as artifacts and marked as missing. Gaps under 20 seconds were filled using cubic spline interpolation, only if the heart rate value before and after the gap differed by less than 50 beats per minute.

## Heart Rate Variability Indices

Next, baseline heart rate, average deceleration capacity, standard deviation, skewness, and sample entropy were calculated over the heart rate signals sampled at 1 Hz. These indices were selected to capture complementary aspects of autonomic regulation, including time-domain, statistical-domain, and nonlinear-domain indices. Baseline heart rate was determined using the weighted median filter method described by Boudet et al. 16 This method assigns weights to the heart rate signal based on the likelihood that a given value represents the true baseline or an acceleration/deceleration event. The likelihood is estimated by analyzing the signal's stability at low frequencies and gradually trimming the data to isolate the baseline. The average deceleration capacity was determined using a phase-rectified signal averaging–based approach, described by Fanelli et al. 17 Deceleration-related anchors are identified in the interval series, locally aligned, and averaged to quantify the mean deceleration response over the recording. The standard deviation of the heart rate time series was computed as the square root of the variance of heart rate values, and skewness was calculated as the third central moment normalized by the cube of the standard deviation. Sample entropy was calculated using the method described by Richman and Moorman, 18 as the negative natural logarithm of the conditional probability that similar patterns of length m remain similar at length m + 1 within tolerance r. Definitions of the heart rate indices are provided in Table 1 . Indices were computed over 1-minute or 5-minute blocks for the entire monitoring period. For fetal heart rate, block values were summarized per day using the median value, while for neonatal heart rate, block values were summarized per hour using the median value.

**Table 1:** Heart rate indices definitions (definitions of the indices are provided, as well as the signal length over which they are calculated and the signal source used)

Index	Definition	Block length	Signal source
Baseline	The mean level of the heart rate when accelerations and decelerations are excluded	1 minute	HR value (1 Hz)
Average deceleration capacity	Integral measure of all periodic deceleration-related oscillations	5 minutes	HR value (1 Hz)
Standard deviation	Standard deviation of consecutive heart rate samples	1 minute	HR value (1 Hz)
Skewness	Measure of the deviation of symmetry of the heart rate distribution	5 minutes	HR value (1 Hz)
Sample entropy	Measure of the regularity of the heart rate	5 minutes	HR value (1 Hz)

## Statistical Analysis

Statistical analysis was performed with SAS (version 9.4, SAS Institute, Cary, North Carolina, United States). Descriptive statistics were used to determine the baseline characteristics. Interrupted time series analysis was performed to evaluate heart rate trends in the week prior to birth, in the week after birth, and the change between these periods. 19 A generalized estimating equation (GEE) model was fitted to the heart rate data in the week before birth and to the heart rate data in the week after birth, providing estimates of second-order nonlinear trends and the change in trend after birth. This approach accounted for the correlation inherent in multiple measurements from the same subject. In addition, subgroup analyses were conducted to explore potential differences between sex, birth weight, mode of delivery, Apgar score at 5 minutes, umbilical cord pH, and neonatal medication. A difference-in-differences analysis was employed to assess whether changes in heart rate differed across these subgroups. Statistical significance was set at *p* < 0.05.

## Results

**Fig. 1:**
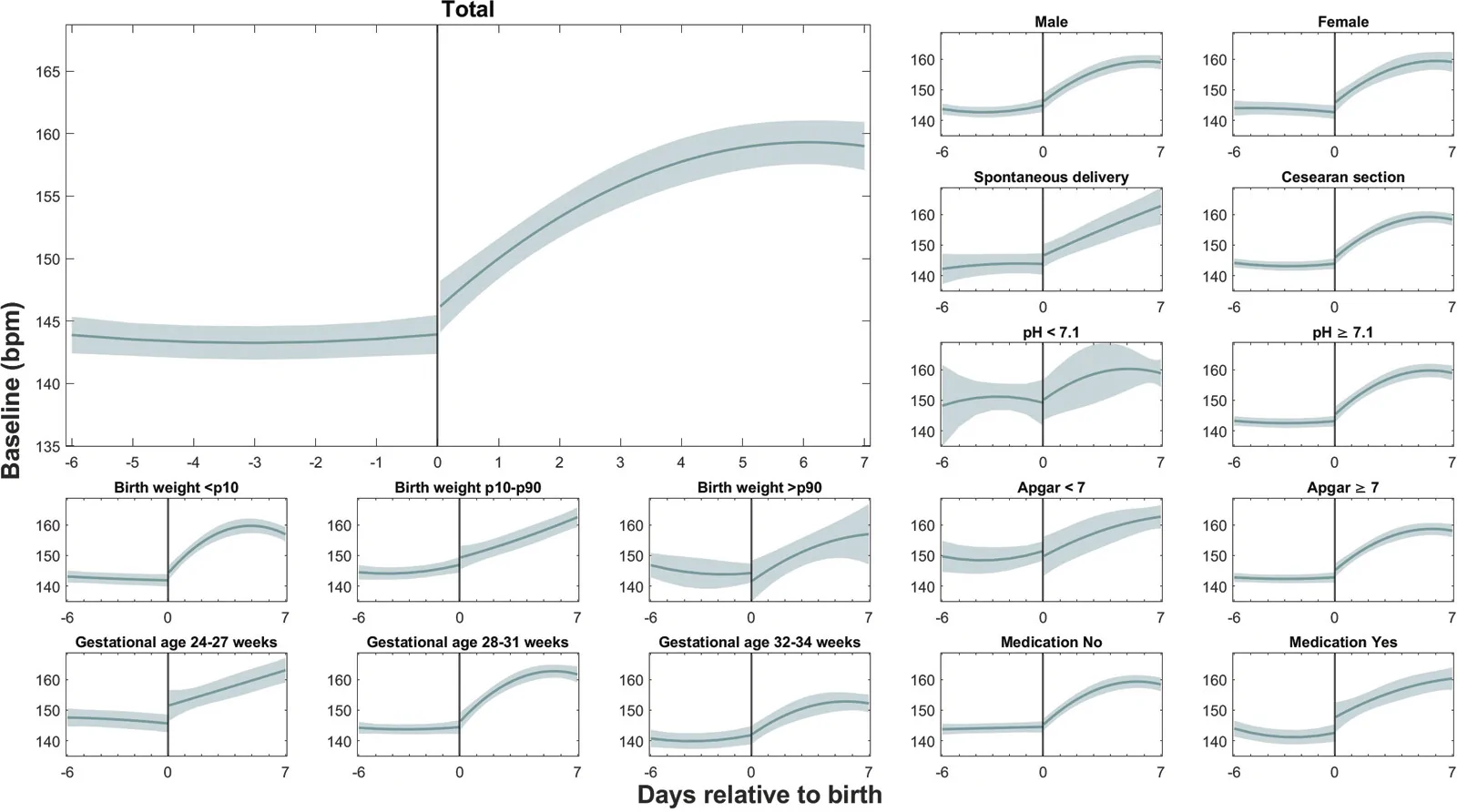
Fitted models for baseline heart rate in the week prior and after birth, along with the 95% confidence intervals for the total group and subanalyses.

**Fig. 2:**
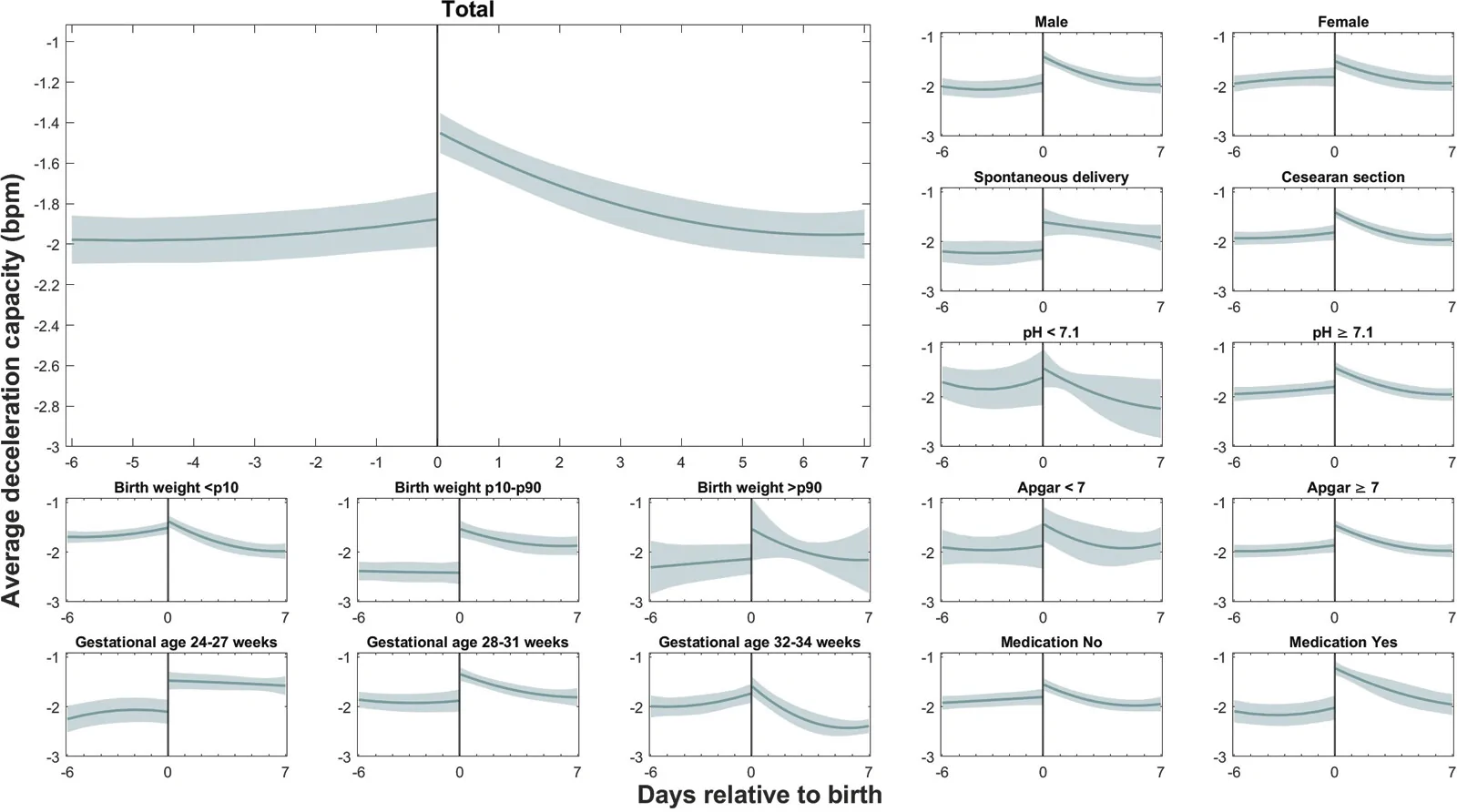
Fitted models for average deceleration capacity in the week prior and after birth, along with the 95% confidence intervals for the total group and subanalyses.

**Fig. 3:**
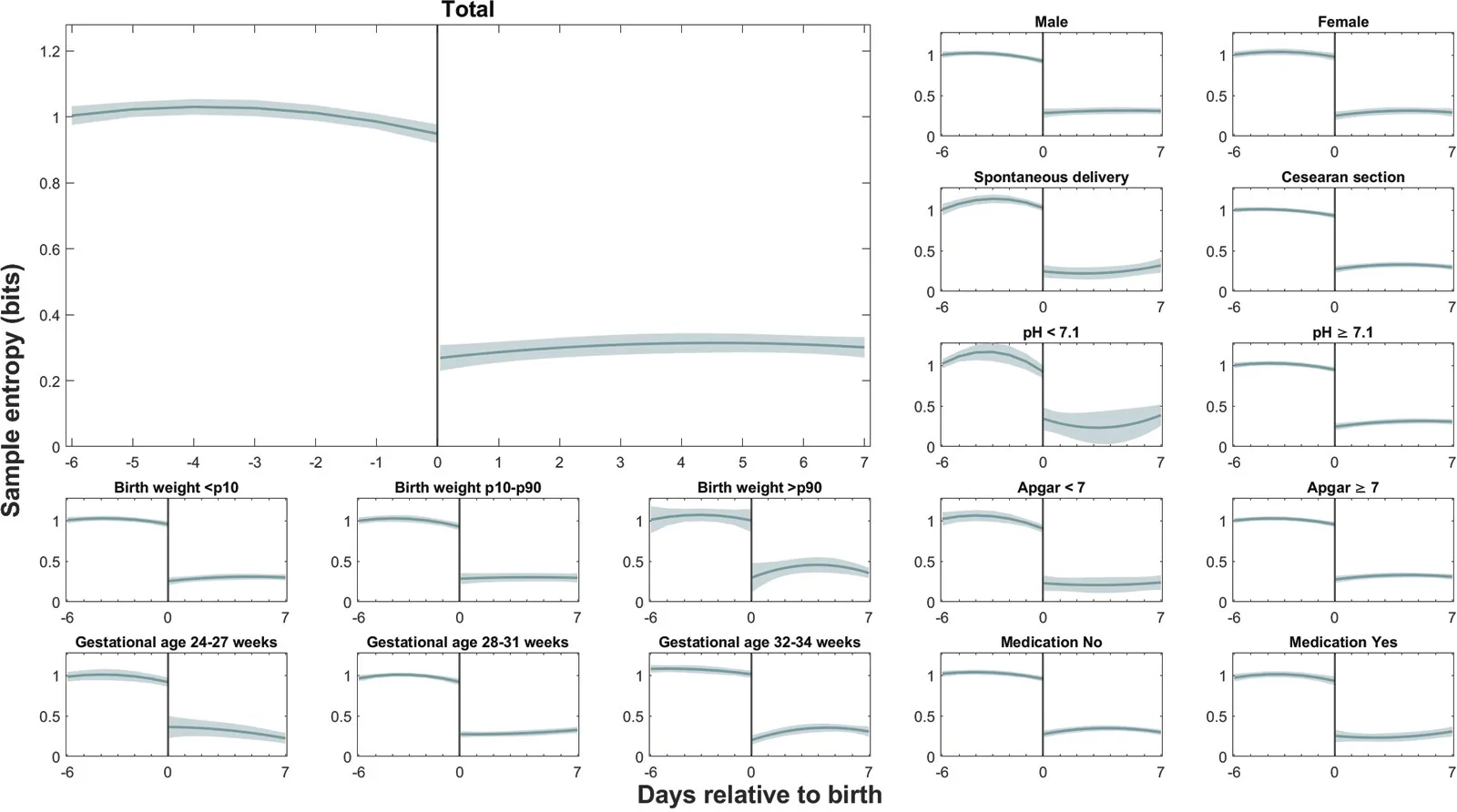
Fitted models for sample entropy in the week prior and after birth, along with the 95% confidence intervals for the total group and subanalyses.

**Fig. 4:**
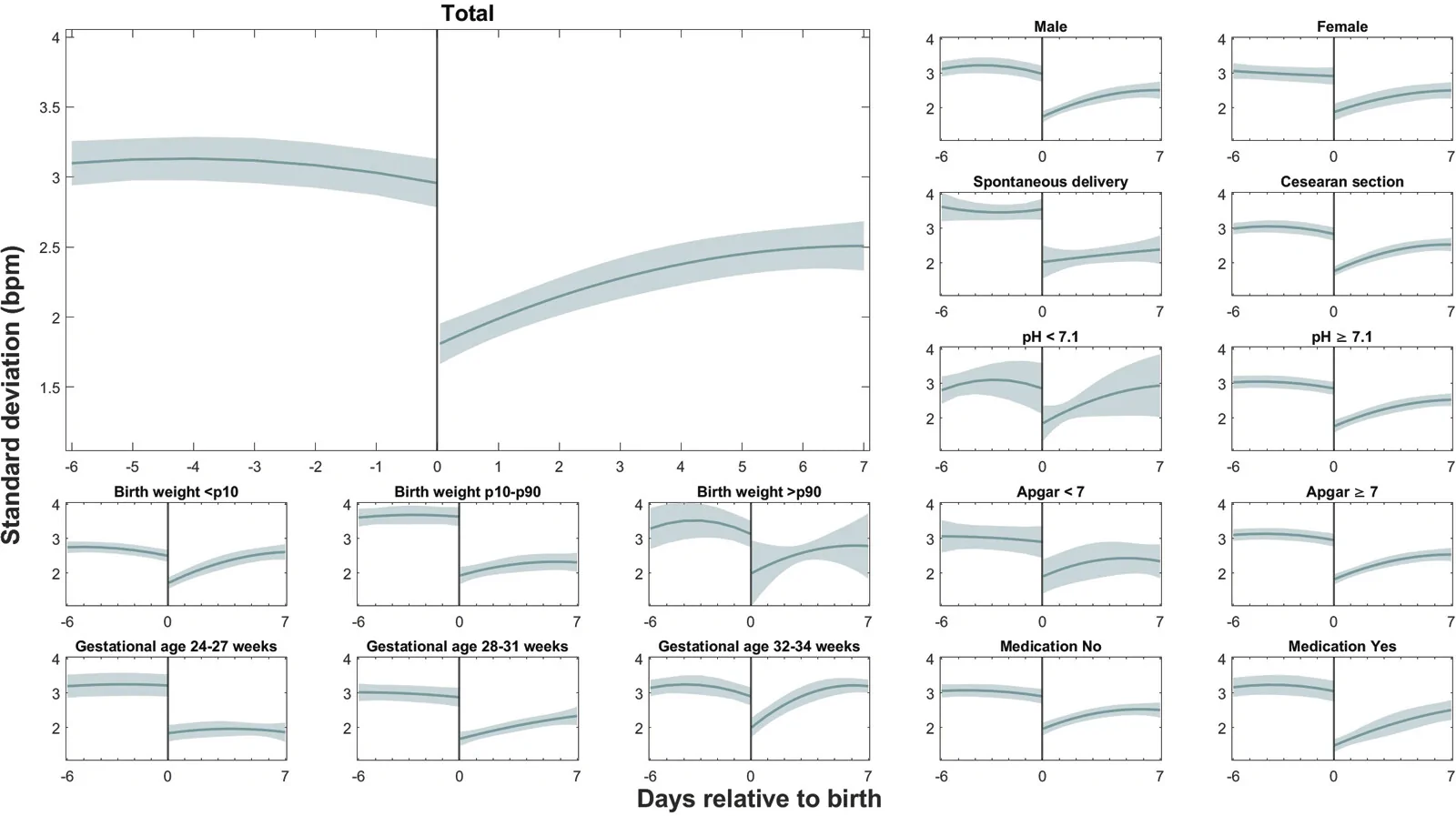
Fitted models for standard deviation in the week prior and after birth, along with the 95% confidence intervals for the total group and subanalyses.

**Fig. 5:**
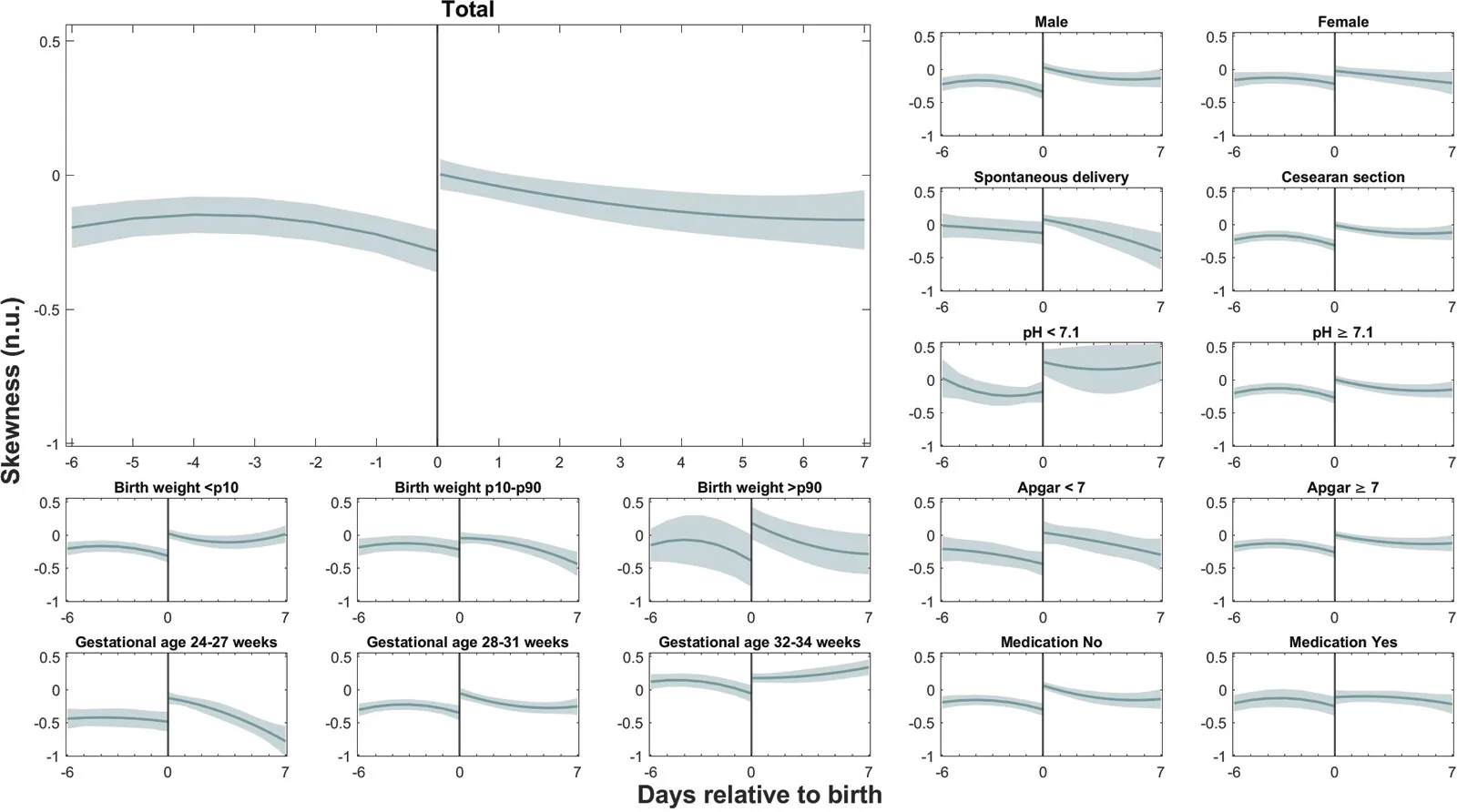
Fitted models for skewness in the week prior and after birth, along with the 95% confidence intervals for the total group and subanalyses.

A total of 123 patients with daily fetal heart rate monitoring in the week prior to birth and continuous neonatal heart rate monitoring during the first week after birth were included. Baseline characteristics for sex, gestational age, birth weight, mode of delivery, Apgar score at 5 minutes, umbilical cord pH, and medication use are presented in Table 2 . Figs. 1 – 5 show the fitted models for the week before and after birth, along with the 95% confidence intervals for the total cohort and subgroup analyses for baseline, average deceleration capacity, sample entropy, standard deviation, and skewness.

**Table 2:** Baseline characteristics (baseline characteristics for sex, gestational age, birth weight, mode of delivery, Apgar score at 5 minutes, pH, and neonatal medication administration)

Category	Subgroup	Number (%)
Sex	Male	67 (54)
	Female	56 (46)
Gestational age (wk)	24–27	25 (20)
	28–31	74 (60)
	32–34	24 (20)
Birth weight percentile	<p10	71 (58)
	p10–p90	46 (37)
	>p90	6 (5)
Mode of delivery	Spontaneous vaginal	20 (16)
	Cesarean section	103 (84)
Apgar 5 minutes	<7	15 (12)
	≥7	104 (85)
Umbilical cord pH	<7.1	5 (4)
	≥7.1	92 (75)
Medication	No	83 (67)
	Yes	40 (33)

In the total cohort, baseline heart rate, average deceleration capacity, and skewness increased significantly after birth (2.23 bpm, CI = 0.36–4.10, *p* < 0.05; 0.43 bpm, CI = 0.29–0.56, *p* < 0.001; 0.29 nu, CI = 0.20–0.37, *p* < 0.001), while sample entropy and standard deviation decreased significantly (−0.68 bits, CI = −0.73 to −0.63, *p* < 0.001; −1.15 bpm, CI = −1.32 to −0.98, *p* < 0.001). The step change of the heart rate indices after birth for the total cohort and subgroups are presented in Supplementary Material Table S1 (available in the online version).

Table 3 presents the results of the difference-in-differences analysis for the subgroups, assessing the step change in heart rate. The postnatal increase in skewness was greater in males, whereas decreases in sample entropy and standard deviation were attenuated in neonates delivered by cesarean section. Exposure to medication with a known influence on the heart rate was associated with larger postnatal increase in baseline and average deceleration capacity and greater decrease in variability metrics. Furthermore, extreme birth weights and gestational age influenced the magnitude of fetal-to-neonatal changes.

**Table 3:** Difference-in-differences estimates for postnatal step change per heart rate index and subgroup

Subgroup	Heart rate index				
	Baseline	Average deceleration capacity	Sample entropy	Standard deviation	Skewness
Sex (female–male)	1.88 (1.90)	−0.21 (0.14)	−0.09 (0.05)	0.20 (0.17)	−0.18 (0.08) a
Delivery mode (CS–spontaneous vaginal delivery)	−0.70 (1.95)	−0.16 (0.16)	0.12 (0.06) a	0.46 (0.23) a	0.10 (0.10)
Umbilical cord pH (pH ≥ 7.1–pH < 7.1)	0.14 (3.87)	0.19 (0.20)	−0.12 (0.11)	−0.10 (0.25)	−0.18 (0.17)
Apgar 5 minutes (Apgar ≥ 7–Apgar < 7)	4.05 (3.50)	−0.04 (0.28)	−4.6e ^−3^ (0.07)	−0.14 (0.27)	−0.21 (0.12)
Medication (medication–no medication)	4.34 (2.19) a	0.54 (0.14) c	1.48e ^−3^ (0.06)	−0.63 (0.18) c	−0.22 (0.09) a
Birth weight (BW p10–p90–BW < p10)	−0.13 (2.19)	0.75 (0.14) c	0.06 (0.06)	−0.92 (0.17) c	−0.16 (0.09)
Birth weight (BW > p90–BW < p10)	−5.07 (2.91)	0.47 (0.25)	2.12e ^–3^ (0.05)	−0.35 (0.42)	0.23 (0.17)
Birth weight (BW > p90–BW p10–p90)	−4.93 (3.33)	−0.28 (0.27)	−0.06 (0.05)	0.57 (0.43)	0.39 (0.18) a
Gestational age (28 to 31–24 to 27 weeks)	−4.30 (2.71)	−0.13 (0.13)	−0.12 (0.08)	0.21 (0.18)	−0.06 (0.11)
Gestational age (32 to 34–24 to 27 weeks)	−5.21 (2.99)	−0.58 (0.19) b	−0.28 (0.09) b	0.58 (0.25) a	−0.15 (0.13)
Gestational age (32 to 34–28 to 31 weeks)	−0.90 (2.11)	−0.45 (0.19) a	−0.16 (0.05) b	0.37 (0.24)	−0.09 (0.10)

a *p* < 0.05.

b *p* < 0.01.

c *p* < 0.001.

In the total cohort, the slope of the baseline heart rate changed significantly after birth, with an increase in the linear effect (3.95 bpm, CI = 2.40–5.50, *p* < 0.001) and a decrease in the quadratic effect (−0.43 bpm, CI = −0.60 to 0.27, *p* < 0.001). The average deceleration capacity showed a significant decrease in the linear effect of the slope change (−0.20 bpm, CI = −0.29 to −0.11, *p* < 0.001). Sample entropy and standard deviation both showed significant increase in the linear slope effects after birth (0.06 bits, CI = 0.04–0.09, *p* < 0.001; 0.29 bpm, CI = 0.16–0.41, *p* < 0.001; respectively). For skewness, the quadratic slope effect significantly increased following birth (0.01 nu, CI = 0.01–0.02, *p* < 0.05). The slope change of the interrupted time series analysis for the total cohort and subgroups is presented in Supplementary Material Table S2 (available in the online version). Difference-in-differences analyses showed significant interactions between fetal-to-neonatal slope changes and delivery mode, umbilical cord pH, birth weight, and gestational age ( Supplementary Material Table S3 , available in the online version).

## Discussion

This study explored trends in fetal and neonatal heart rate data from 1 week before birth to 1 week after birth in fetuses and neonates admitted to a tertiary care hospital and born preterm. Additionally, we explored heart rate trends across different subgroups categorized by gestational age at birth, sex, birth weight, mode of delivery, Apgar score at 5 minutes, umbilical cord pH, and neonatal medication. Our findings provide unique insights into heart rate frequency and variability trends during the period around birth. It highlights the dynamic physiological adaptation that occurs during the transition from intrauterine to extrauterine life in preterm infants.

The observed postnatal step and slope changes in the heart rate frequency and variability indices reflect the changes in autonomic regulation required for adapting the cardiovascular, respiratory, thermoregulatory, and metabolic systems. 20 Immediately after birth, the sympathetic nervous system predominates, increasing heart rate frequency and decreasing heart rate variability. 21 This is in line with the step changes we observed postnatally for the heart rate indices, including an increased baseline and decreased variability measures reflected by a smaller average deceleration capacity, a less right-skewed heart rate distribution, lower standard deviation, and lower sample entropy. It is suggested that a surge in circulating catecholamines and the neonatal stress response occurring after birth increases systematic autonomic system activity. 22 23 Activation of the sympathetic autonomic system increases heart rate, cardiac output, and blood pressure, among other effects. This helps with regulating circulation, oxygen delivery, glucose metabolism, and thermogenesis. 21 In the days following birth, the parasympathetic nervous system becomes more active, slowing the increase in heart rate frequency and increase in the heart rate variability, aligning with our findings.

Subgroup analyses provided insights into how maternal and neonatal factors influence autonomic adaptation. Delivery mode emerged as a significant factor, with vaginally delivered neonates showing greater postnatal autonomic adjustments than those born by cesarean section, reflecting stronger physiological stress and faster cardiovascular adaptation. These findings are consistent with those of a previous study reporting higher cardiovagal modulation in spontaneous vaginal deliveries without analgesia when compared with deliveries by caesarean section. 24 Neonates receiving medication exhibited more pronounced increase in baseline heart rate and decrease in variability, indicating a potentially delayed or suboptimal autonomic adaptation. Extreme birth weights were associated with altered heart rate dynamics, suggesting suboptimal autonomic adaptation. This is in line with a previous study that found low birth weight linked to lower total power and parasympathetic activity. 25

Our findings highlight the potential clinical utility of monitoring trends in heart rate and heart rate variability. Prospectively, continuous assessment of these trends may allow for earlier detection of subtle physiological deviations that precede clinical deterioration. By providing a higher density of longitudinal data, trend monitoring could inform predictive models, facilitate risk stratification, and support timely clinical decision-making, including the initiation of closer observation or escalation of care. These applications underscore the value of integrating trend-based analyses into routine neonatal monitoring. In addition, our results provide further insight into the physiological processes involved in the fetal-to-neonatal transition, particularly in preterm infants, who remain at increased risk for adverse outcomes. Future research should focus on including a more heterogeneous population and adding more clinical variables to increase the generalizability of findings. Investigating the discriminative capacity of heart rate indices may also improve the early detection and prediction of health risks in fetuses and neonates, allowing for more precise identification and targeted interventions aimed at improving health outcomes.

## Strengths and Limitations

One of the key strengths of this study lies in its longitudinal design. The repeated heart rate measures during both fetal and neonatal period allows for a trend analysis of the heart rate dynamics. The inclusion of multiple heart rate indices alongside several patient characteristics provides a comprehensive insight into the heart rate frequency and variability trends for specific patient groups and potential clinical relevance in this unique cohort. The study has also limitations. Selection bias may have influenced the results, as the cohort was drawn from a tertiary hospital and included only patients with available heart rate data for both the week before and the week after birth. Additionally, the lack of data on clinical outcomes, such as obstetric complications and long-term neonatal outcomes, restricts the scope of our conclusions and their applicability to broader clinical contexts.

## Conclusion

In conclusion, this study provides heart rate frequency and variability trends during the week before and after birth in preterm neonates, offering valuable insights into cardiovascular adaptation during this critical transition. These findings contribute to a better understanding of heart rate dynamics in early life and may help inform future research on fetal and neonatal monitoring and clinical management.
