# Supplementary material for: Fetal and Neonatal Heart Rate Trends in Preterm Delivery: A Clinical Study from the Week before to the Week after Birth
Source: Am J Perinatol. 2026 Feb 23;43(12):1613–20. doi: 10.1055/a-2806-2923 (PMC13503025; doi:10.1055/a-2806-2923)
Supplement: Supplementary file 1 [file 10-1055-a-2806-2923_29438220.pdf]

Supplementary Table S1. Step change of interrupted time series analysis.

	Baseline	Average deceleration capacity	Sample entropy	Standard deviation	Skewness
**Total**	2,23* (0,36 – 4,10)	0,43** (0,29 – 0,56)	-0,68** (-0,73 – -0,63)	-1,15** (-1,32 –- 0,98)	0,29** (0,20 – 0,37)
**Male**	1,37 (-1,20 – 3,94)	0,52** (0,32 – 0,72)	-0,64** (-0,71 – -0,57)	-1,24** (-1,47 – -1,00)	0,37** (0,25 – 0,48)
**Female**	3,26* (0,57 – 5,94)	0,31* (0,13 – 0,50)	-0,73** (-0,80 – -0,66)	-1,04** (-1,29 – -0,79)	0,19* (0,08 – 0,30)
**Spontaneous delivery**	2,81 (-0,37 – 5,98)	0,56** (0,29 – 0,82)	-0,78** (-0,89 – -0,68)	-1,53** (-1,93 – -1,13)	0,30** (0,21 – 0,40)
**Cesarean section**	2,12 (-0,02 – 4,26)	0,40** (0,24 – 0,56)	-0,66** (-0,72 – -0,60)	-1,07** (-1,26 – -0,89)	0,20* (0,03 – 0,37)
**pH <7.1**	0,97 (-6,27 – 8,22)	0,18 (-0,17 – 0,53)	-0,58** (-0,78 – -0,38)	-1,01** (-1,45 – -0,56)	0,45* (0,13 – 0,76)
**pH >=7.1**	2,33* (0,08 – 4,58)	0,37** (0,22 – 0,53)	-0,70** (-0,76 – -0,65)	-1,09** (-1,29 – -0,90)	0,27** (0,18 – 0,36)
**Apgar < 7**	-1,61 (-8,18 – 4,95)	0,43 (-0,09 – 0,96)	-0,67** (-0,81 – -0,54)	-1,00** (-1,50 – -0,50)	0,47** (0,26 – 0,69)
**Apgar >=7**	2,44* (0,49 – 4,38)	0,40** (0,26 – 0,53)	-0,68** (-0,74 – -0,62)	-1,14** (-1,32 – -0,95)	0,26** (0,17 – 0,35)
**No medication**	0,82 (-1,19 – 2,82)	0,25* (0,09 – 0,40)	-0,68** (-0,74 – -0,62)	-0,94** (-1,14 – -0,75)	0,36** (0,27 – 0,45)
**Medication**	5,16* (1,36 – 8,96)	0,79** (0,55 – 1,03)	-0,68** (-0,78 – -0,58)	-1,57** (-1,87 – -1,28)	0,13 (-0,02 – 0,29)
**Birth weight <p10**	2,53* (0,50 – 4,55)	0,13 (0,00 – 0,26)	-0,70** (-0,77 – -0,63)	-0,79** (-0,97 – -0,60)	0,33** (0,23 – 0,44)
**Birth weight p10-p90**	2,39 (-1,40 – 6,19)	0,88** (0,63 – 1,13)	-0,64** (-0,73 – -0,55)	-1,71** (-1,98 – -1,43)	0,17* (0,03 – 0,31)
**Birth weight >p90**	-2,54 (-7,86 – 2,78)	0,60* (0,13 – 1,06)	-0,70** (-0,77 – -0,64)	-1,14* (-1,93 – -0,34)	0,57* (0,25 – 0,89)
**Gestational age 24-27 weeks**	5,84* (1,07 – 10,60)	0,62** (0,45 – 0,80)	-0,55** (-0,70 – -0,41)	-1,38** (-1,65 – -1,11)	0,36** (0,18 – 0,53)
**Gestational age 28-31 weeks**	1,53 (-0,82 – 3,88)	0,49** (0,31 – 0,67)	-0,67** (-0,73 – -0,61)	-1,18** (-1,41 – -0,95)	0,29** (0,18 – 0,40)
**Gestational age 32-34 weeks**	0,63 (-2,78 – 4,03)	0,04 (-0,29 – 0,37)	-0,83** (-0,92 – -0,74)	-0,81** (-1,22 – -0,39)	0,20* (0,03 – 0,38)

*p<0.05, **p<0.001

Supplementary Table S2. Slope change of interrupted time series analysis.

	Baseline		Average deceleration capacity		Sample entropy		Standard deviation		Skewness	
	Linear	Quadratic	Linear	Quadratic	Linear	Quadratic	Linear	Quadratic	Linear	Quadratic
**Total**	3.95**<br>(2.40 – 5.49)	-0.43**<br>(-0.60 – -0.27)	0.20**<br>(-0.29 – -0.11)	0.01<br>(-3.23e^{-3} – 0.02)	0.06**<br>(0.04 – 0.09)	3.35e^{-3}<br>(-4.80e^{-4} – 7.19e^{-3})	0.29**<br>(0.16 – 0.41)	-4.35e^{-3}<br>(-0.02 – 0.01)	0.02<br>(-0.03 – 0.08)	0.01*<br>(5.31e^{-3} – 0.02)
**Male**	3.02*<br>(1.15 – 4.88)	-0.53**<br>(-0.73 – -0.33)	-0.25*<br>(-0.38 – -0.12)	3.23e^{-3}<br>(-0.015 – 0.021)	0.06*<br>(0.03 – 0.10)	4.55e^{-3}<br>(-9.20e^{-4} – 0.01)	0.37**<br>(0.19 – 0.55)	2.54e^{-3}<br>(-0.02 – 0.02)	0.02<br>(-0.05 – 0.09)	0.02*<br>(7.52e^{-3} – 0.03)
**Female**	5.06**<br>(2.53 – 7.59)	-0.32*<br>(-0.58 – -0.06)	-0.14*<br>(-0.24 – -0.03)	0.01*<br>(1.38e^{-3} – 0.03)	0.06*<br>(0.03 – 0.10)	1.94e^{-3}<br>(-3.33e^{-3} – 0.01)	0.19*<br>(0.02 – 0.35)	-0.01<br>(-0.03 – 4.73e^{-3})	0.02<br>(-0.06 – 0.10)	6.57e^{-3}<br>(-4.10e^{-3} – 0.02)
**Spontaneous delivery**	2.76<br>(-0.17 – 5.69)	0.05<br>(-0.24 – 0.35)	-0.08<br>(-0.26 – 0.10)	-5.12e^{-3}<br>(-0.03 – 0.02)	0.06<br>(-0.01 – 0.12)	0.02**<br>(0.01 – 0.03)	-7.66e^{-3}<br>(-0.31 – 0.30)	-0.02<br>(-0.05 – 0.02)	-0.03<br>(-0.15 – 0.09)	-2.86e^{-3}<br>(-0.02 – 0.01)
**Cesarean section**	4.18**<br>(2.42 – 5.93)	-0.53**<br>(-0.71 – -0.35)	-0.22**<br>(-0.32 – -0.13)	0.01<br>(-1.96e^{-3} – 0.02)	0.06**<br>(0.04 – 0.10)	3.80e^{-4}<br>(-3.711e^{-3} – 4.47e^{-3})	0.34**<br>(0.21 – 0.48)	-2.22e^{-3}<br>(-0.02 – 0.01)	0.03<br>(-0.03 – 0.09)	0.02*<br>(7.49e^{-3} – 0.03)
**pH <7.1**	5.487<br>(-1.33 – 12.28)	-0.12<br>(-1.11 – 0.87)	-0.34<br>(-0.79 – 0.11)	-0.01<br>(-0.04 – 0.02)	0.08<br>(-0.09 – 0.24)	0.03**<br>(0.03 – 0.04)	0.45*<br>(7.89e^{-3} – 0.90)	0.01<br>(-0.04 – 0.06)	-0.13<br>(-0.33 – 0.07)	-7.89e^{-3}<br>(-0.04 – 0.03)
**pH >=7.1**	4.63**<br>(2.75 – 6.52)	-0.53**<br>(-0.72 – -0.34)	-0.20**<br>(-0.30 – -0.10)	0.01<br>(-2.34e^{-3} – 0.02)	0.07**<br>(0.04 – 0.10)	2.61e^{-3}<br>(-1.68e^{-3} – 0.01)	0.30**<br>(0.15 – 0.44)	-4.24e^{-3}<br>(-0.02 – 0.01)	0.02<br>(-0.04 – 0.08)	0.02**<br>(0.01 – 0.03)
**Apgar 5 min < 7**	1.28<br>(-1.43 – 3.99)	-0.39<br>(-0.90 – 0.12)	-0.26*<br>(-0.492 – -0.022)	0.01<br>(-0.02 – 0.04)	0.07<br>(-3.38e^{-3} – 0.14)	0.01*<br>(6.20e^{-4} – 0.02)	0.27<br>(-0.05 – 0.59)	-0.02<br>(-0.05 – 0.02)	0.03<br>(-0.17 – 0.23)	2.59e^{-3}<br>(-0.02 – 0.02)
**Apgar 5 min >=7**	4.33**<br>(2.56 – 6.10)	-0.46**<br>(-0.63 – -0.29)	-0.20**<br>(-0.29 – -0.11)	0.01<br>(-4.31e^{-3} – 0.02)	0.06**<br>(0.03 – 0.09)	2.26e^{-3}<br>(-1.78e^{-3} – 6.30e^{-3})	0.29**<br>(0.16 – 0.43)	-4.14e^{-3}<br>(-0.02 – 0.01)	0.02<br>(-0.03 – 0.08)	0.01*<br>(5.52e^{-3} – 0.02)
**No medication**	4.95**<br>(3.30 – 6.60)	-0.45**<br>(-0.63 – -0.27)	-0.18*<br>(-0.28 – -0.07)	0.01*<br>(9.65e^{-4} – 0.03)	0.08**<br>(0.05 – 0.11)	-4.00e^{-5}<br>(-4.69e^{-3} – 4.60e^{-3})	0.27*<br>(0.12 – 0.42)	-8.59e^{-3}<br>(-0.03 – 0.01)	-0.01<br>(-0.07 – 0.06)	0.02*<br>(7.06e^{-3} – 0.03)
**Medication**	1.86<br>(-1.33 – 5.06)	-0.40*<br>(-0.73 – -0.06)	-0.25*<br>(-0.40 – -0.09)	-3.99e^{-3}<br>(-0.03 – 0.02)	0.03<br>(-0.03 – 0.09)	0.01*<br>(4.20e^{-3} – 0.02)	0.31*<br>(0.10 – 0.52)	4.43e^{-3}<br>(-0.02 – 0.03)	0.09<br>(4.90e^{-4} – 0.18)	6.67e^{-3}<br>(-7.96e^{-3} – 0.02)
**Birth weight <p10**	6.42**<br>(4.46 – 8.37)	-0.65**<br>(-0.87 – -0.44)	-0.25**<br>(-0.35 – -0.16)	0.01<br>(-4.30e^{-3} – 0.02)	0.06*<br>(0.02 – 0.10)	2.52e^{-3}<br>(-2.93e^{-3} – 0.01)	0.34**<br>(0.21 – 0.46)	-0.01<br>(-0.02 – 0.01)	1.10e^{-4}<br>(-0.06 – 0.06)	0.02*<br>(0.01 – 0.03)
**Birth weight p10-p90**	0.21<br>(-2.22 – 2.63)	-0.10<br>(-0.35 – 0.15)	-0.11<br>(-0.286 – 0.063)	0.01<br>(-0.02 – 0.03)	0.06*<br>(0.02 – 0.10)	5.59e^{-3}*<br>(5.00e^{-5} – 0.01)	0.18<br>(-0.08 – 0.44)	-0.01<br>(-0.04 – 0.03)	0.06<br>(-0.05 – 0.16)	6.14e^{-4}<br>(-0.01 – 0.01)
**Birth weight >p90**	3.36*<br>(0.74 – 5.99)	-0.41<br>(-0.82 – 6.8e^{-3})	-0.22<br>(-0.442 – 0.003)	0.02<br>(-0.03 – 0.06)	0.13<br>(-0.03 – 0.28)	-3.28e^{-3}<br>(-0.01 – 8.43e^{-3})	0.50*<br>(0.06 – 0.93)	0.01<br>(-0.04 – 0.07)	0.03<br>(-0.15 – 0.21)	0.03*<br>(3.40e^{-3} – 0.05)
**Gestational age 24-27 weeks**	2.16<br>(-0.75 – 5.07)	0.04<br>(-0.30 – 0.38)	0.03<br>(-0.10 – 0.16)	0.01<br>(-4.91e^{-3} – 0.03)	0.04<br>(-0.03 – 0.12)	3.47e^{-3}<br>(-3.00e^{-3} – 0.01)	0.09<br>(-0.08 – 0.27)	-4.38e^{-3}<br>(-0.02 – 0.02)	-0.01<br>(-0.14 – 0.13)	-3.23e^{-3}<br>(-0.02 – 0.02)
**Gestational age 28-31 weeks**	5.19**<br>(3.07 – 7.32)	-0.59**<br>(-0.79 – -0.39)	-0.21*<br>(-0.33 – -0.09)	1.36e^{-3}<br>(-0.01 – 0.02)	0.05*<br>(0.02 – 0.08)	5.79e^{-3}*<br>(9.30e^{-4} – 0.01)	0.26*<br>(0.09 – 0.44)	2.66e^{-3}<br>(-0.02 – 0.02)	4.05e^{-3}<br>(-0.07 – 0.07)	0.02*<br>(0.01 – 0.03)
**Gestational age 32-34 weeks**	1.96<br>(-0.97 – 4.88)	-0.45*<br>(-0.84 – -0.06)	-0.39**<br>(-0.56 – -0.23)	0.03*<br>(1.88e^{-4} – 0.05)	0.12*<br>(0.05 – 0.19)	-4.16e^{-3}<br>(-0.01 –5.76e^{-3})	0.56**<br>(0.32 – 0.79)	-0.03<br>(-0.06 – 3.33e^{-3})	0.11*<br>(0.04 – 0.18)	0.01*<br>(1.92e^{-4} – 0.03)

*p<0.05, **p<0.001

Supplementary Table S3. Difference-in-Differences estimates for postnatal slope change per heart rate index and subgroup.

Subgroup	Heart rate index				
	Baseline	Average deceleration capacity	Sample entropy	Standard deviation	Skewness
**Sex**<br>(female – male)	0.21<br>(-0.12 – 0.54)	0.01<br>(-0.02 – 0.03)	$2.62e^{-3}$<br>(-0.01 – $4.98e^{-3}$)	-0.02<br>(-0.04 – 0.01)	-0.01<br>(-0.03 – $3.24e^{-3}$)
**Delivery mode**<br>*(CS – spontaneous vaginal delivery)*	-0.58***<br>(-0.92 – -0.23)	0.02<br>(-0.01 – 0.04)	-0.2***<br>(-0.03 – -0.01)	0.01<br>(-0.02 – 0.05)	0.02*<br>($2.81e^{-3}$ – 0.04)
**Umbilical cord pH**<br>*(pH ≥7.1 – pH < 7.1)*	-0.41<br>(-1.42 – 0.60)	0.02<br>(-0.01 – 0.05)	-0.03***<br>(-0.04 – -0.02)	-0.02<br>(-0.07 – 0.03)	0.03<br>(-0.01 – 0.06)
**Apgar 5 min**<br>*(Apgar ≥ 7 – Apgar < 7)*	-0.067<br>(-0.61 – 0.47)	$-4.73e^{-3}$<br>(-0.04 – 0.03)	-0.01<br>(-0.02 – $2.30e^{-3}$)	0.01<br>(-0.01 – 0.03)	0.01
**Medication**<br>*(Medication – No medication)*	0.06<br>(-0.32 – 0.44)	-0.02<br>(-0.04 – 7.36e-3)	0.01**<br>($2.70e^{-3}$ – 0.02)	0.01<br>(-0.02 – 0.05)	$9.81e^{-3}$<br>(-0.03 – $7.59e^{-3}$)
**Birth weight**<br>*(BW p10-p90 – BW < p10)*	0.55**<br>(0.19 – 0.91)	0.01<br>(–0.02 – 0.04)	$7.37e^{-3}$<br>(-0.01 – 0.0.1)	$5.05e^{-4}$<br>(-0.03 – 0.03)	-0.02<br>(-0.05 – 0.01)
**Birth weight**<br>*(BW > p90 – BW < p10)*	0.30<br>(–0.28 – 0.88)	0.02<br>(–0.02 – 0.06)	-0.01<br>(-0.03 – 0.01)	$6.81e^{-3}$<br>(0.06 – 0.06)	$4.00e^{-4}$<br>(-0.03 – 0.03)
**Birth weight**<br>*(BW > p90 – BW p10-p90)*	-0.31<br>(–0.86 – 0.24)	0.01<br>(–0.03 – 0.05)	-0.01<br>(-0.03 – 0.01)	0.02<br>(-0.05 – 0.09)	0.03<br>(0.00 – 0.06)
**Gestational age**<br>*(28-31 weeks – 24-27 weeks)*	-0.63*<br>(-1.05 – -0.21)	$-9.20e^{-3}$<br>(-0.03 – 0.01)	$2.30e^{-3}$<br>(-0.01 – 0.01)	$0.70e^{-3}$<br>(-0.03 – 0.04)	0.02<br>(-0.00 – 0.05)
**Gestational age**<br>*(32-34 weeks – 24-27 weeks)*	-1.01***<br>(-1.56 – 0.46)	0.03<br>(-0.01 – 0.06)	$-7.50e^{-3}$<br>(-0.02 – 0.01)	-0.03<br>(-0.07 – 0.01)	0.03<br>(-0.00 – 0.05)
**Gestational age**<br>*(32-34 weeks – 28-31 weeks)*	0.14<br>(-0.31 – 0.60)	0.03<br>(-0.01 – 0.06)	-0.1<br>(-0.02 – 0.00)	-0.03<br>(-0.07 -0.01)	$-3.80e^{-3}$<br>(-0.02 – 0.01)

*p<0.05, **p<0.01, ***p<0.001
